# *In Silico* Modeling of Biofilm Formation by Nontypeable Haemophilus influenzae *In Vivo*

**DOI:** 10.1128/mSphere.00254-19

**Published:** 2019-07-31

**Authors:** Jonathan R. Brown, Joseph Jurcisek, Vinal Lakhani, Ali Snedden, William C. Ray, Elaine M. Mokrzan, Lauren O. Bakaletz, Jayajit Das

**Affiliations:** a Battelle Center for Mathematical Medicine, The Research Institute at the Nationwide Children’s Hospital, Columbus, Ohio, USA; b Department of Pediatrics, College of Medicine, the Ohio State University, Columbus, Ohio, USA; c Department of Biophysics Graduate Program, the Ohio State University, Columbus, Ohio, USA; d Center for Microbial Pathogenesis, The Abigail Wexner Research Institute at the Nationwide Children’s Hospital, Columbus, Ohio, USA; e High Performance Computing Center, The Research Institute at the Nationwide Children’s Hospital, Columbus, Ohio, USA; University of Kentucky

**Keywords:** *Chinchilla lanigera*, agent-based model, biofilm, host response, *in silico* modeling, *in vivo*, middle ear, nontypeable *Haemophilus influenzae*, otitis media, pair correlation, parameter estimation, particle swarm

## Abstract

Biofilms formed by nontypeable Haemophilus influenzae (NTHI) bacteria play an important role in multiple respiratory tract diseases. Visual inspection of the morphology of biofilms formed during chronic infections shows distinct differences from biofilms formed *in vitro*. To better understand these differences, we analyzed images of NTHI biofilms formed in the middle ears of Chinchilla lanigera and developed an *in silico* agent-based model of the formation of NTHI biofilms *in vivo*. We found that, as *in vitro*, NTHI bacteria are organized in self-similar patterns; however, the sizes of NTHI clusters *in vivo* are more than 10-fold smaller than their *in vitro* counterparts. The agent-based model reproduced these patterns and suggested that smaller clusters occur due to elimination of planktonic NTHI cells by the host responses. Estimation of model parameters by fitting simulation results to imaging data showed that the effects of several processes in the model change during the course of the infection.

**IMPORTANCE** Multiple respiratory illnesses are associated with formation of biofilms within the human airway by NTHI. However, a substantial amount of our understanding of the mechanisms that underlie NTHI biofilm formation is obtained from *in vitro* studies. Our *in silico* model that describes biofilm formation by NTHI within the middle ears of Chinchilla lanigera will help isolate processes potentially responsible for the differences between the morphologies of biofilms formed *in vivo* versus those formed *in vitro*. Thus, the *in silico* model can be used to glean mechanisms that underlie biofilm formation *in vivo* and connect those mechanisms to those obtained from *in vitro* experiments. The *in silico* model developed here can be extended to investigate potential roles of specific host responses (e.g., mucociliary clearance) on NTHI biofilm formation *in vivo*. The developed computational tools can also be used to analyze and describe biofilm formation by other bacterial species *in vivo*.

## Abstract (precis)

Multiple respiratory illnesses are associated with formation of biofilms within the human airway by NTHI. However, a substantial amount of our understanding of the mechanisms that underlie NTHI biofilm formation is obtained from *in vitro* studies. Our *in silico* model that describes biofilm formation by NTHI within the middle ears of Chinchilla lanigera will help isolate processes potentially responsible for the differences between the morphologies of biofilms formed *in vivo* versus those formed *in vitro*. Thus, the *in silico* model can be used to glean mechanisms that underlie biofilm formation *in vivo* and connect those mechanisms to those obtained from *in vitro* experiments. The *in silico* model developed here can be extended to investigate potential roles of specific host responses (e.g., mucociliary clearance) on NTHI biofilm formation *in vivo*. The developed computational tools can also be used to analyze and describe biofilm formation by other bacterial species *in vivo*.

## INTRODUCTION

Nontypeable Haemophilus influenzae (NTHI) is a major pathogen for a range of polymicrobial diseases in the upper and lower respiratory tract, including otitis media (OM) (1), sinusitis, and exacerbations of chronic obstructive pulmonary disease (COPD) (1). Biofilm formation by NTHI is a hallmark for many of the above diseases (2) where NTHI bacteria organize into complex three-dimensional structures within the local host environment (3–6). NTHI biofilms are supported by an extracellular matrix scaffold (ECM) comprised of a large variety of components that include extracellular DNA, proteins, and polysaccharides (5, 7, 8). Formation of NTHI biofilms has been investigated both *in vitro* and *in vivo* in a chinchilla model of OM (4, 6, 9–11) and in middle ear mucosal biopsy specimens obtained from human patients (3). The NTHI biofilms formed *in vitro* and *in vivo* showed similarities in terms of expression of bacterial oligosaccharides (e.g., lipooligosaccharide) (4, 12), type IV pili (11, 13), and extracellular DNA (4, 5, 8), all of which contribute to biofilm formation.

However, spatial morphologies of the biofilms formed *in vivo* and *in vitro* by the same bacterial pathogen can show distinct differences. Bjarnsholt et al. (14) analyzed the sizes of bacterial aggregates in biofilms formed in parts of the human body in several diseases such as cystic fibrosis, chronic wounds, otitis media, and rhinosinusitis and concluded that the biofilm sizes in chronic infections span a much smaller range (∼50 to 200 μm) than the biofilms formed by these bacterial pathogens *in vitro* where the biofilms can extend over several centimeters. Furthermore, specific morphological features such as mushroom-like structures in biofilms formed by Pseudomonas aeruginosa *in vitro* were absent in biofilms formed by the bacterial pathogen within the host (14, 15). It has been speculated that these differences arise due to the differences between the local environment in the *in vitro* culture and the host, e.g., nutrient-rich culture medium versus nutrient-restricted local host environment (14). Furthermore, the host responses such as mucociliary clearance (16), production of antimicrobial proteins as effectors of innate immunity (17), and nutritional immunity (18), among others partially eliminate bacterial cells in the biofilm. These host responses are absent *in vitro* and can contribute to specific structural differences in biofilms formed *in vivo*.

In order to characterize the spatial structures of biofilms formed by NTHI *in vivo* and determine potential mechanisms that underlie biofilm formation that occurs within a mammalian host, we first quantitatively characterized the spatial patterns of NTHI in confocal laser scanning microscopy (CLSM) images of biofilms formed by NTHI in the middle ears of chinchillas during experimental NTHI-induced OM and then developed a three-dimensional *in silico* agent-based model to describe formation of NTHI biofilms *in vivo*. The spatial patterns were analyzed by two-point pair correlation function, which has been widely used in statistical physics and material science to characterize spatial structures. The correlation length calculated from the correlation function provides an estimation of the characteristic size of the bacterial clusters in the biofilm. The agent-based model is a modification of our *in silico* model developed previously to describe formation of NTHI biofilms *in vitro* (7). The parameters in the agent-based model were estimated by comparing architectural features (as calculated from the pair correlation function) of the *in silico* biofilms with those of CLSM images obtained from NTHI biofilms formed in the chinchilla middle ears (4). A variety of *in silico* models based on agent-based models (19), partial differential equations (20–22), and particle-based simulations (23–25) have been developed previously to describe biofilm formation of different bacterial species such as P. aeruginosa and Vibrio cholerae. However, the majority of these models dealt with biofilm formation *in vitro*, and thus cannot be applied directly to describe biofilm formation *in vivo*.

Our analysis of the CLSM images for the NTHI biofilms formed *in vivo* found that NTHI bacterial cells are organized in fractal structures within the biofilms. This pattern is similar to that found in NTHI biofilms formed *in vitro* (7); however, the size of the NTHI clusters, as defined by the correlation length, *in vivo* are much smaller than their *in vitro* counterparts. The agent-based model statistically reproduced the spatial organization of NTHI bacterial cells in the biofilm formed *in vivo*. Our modeling results suggest that the dispersion of biofilm-associated NTHI bacterial cells induced by quorum sensing and the elimination of the planktonic or nonadherent NTHI bacterial cells by mechanisms not present *in vitro* such as the host response play key roles in giving rise to the smaller NTHI spatial clusters *in vivo*. The model parameter values estimated for NTHI biofilms 4 days and 11 days after challenge indicate changes in the rate at which planktonic bacteria are eliminated and NTHI quorum sensing during the course of the infection.

## RESULTS

### Quantitative characterization of NTHI biofilms formed *in vivo*.

**FIG 1:**
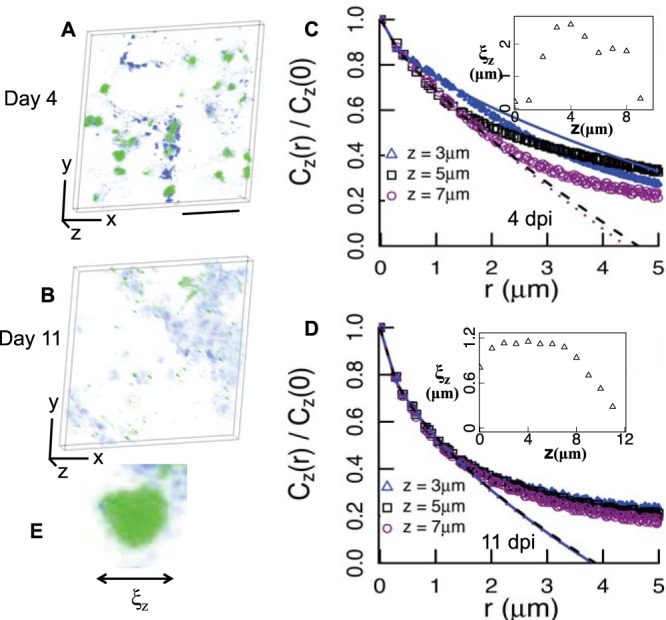
Quantitative characterization of spatial organization of NTHI in biofilms formed *in vivo* shows a fractal structure. (A and B) Organization of NTHI (green) and DNA strands (blue) in three-dimensional (3D) renderings derived from CLSM images for biofilm section of 10-μm thickness (*z* direction) and a cross-section of 148 μm (*x* direction) by 148 μm (*y* direction) obtained from the chinchilla middle ear at day 4 (A) and day 11 (B) postchallenge. The black bar indicates 50 μm. (C) Variation of *C_z_*(*r*)/*C_z_*(0) with *r* for the spatial patterns of NTHI in the CLSM images in panel A. The *z* values indicate the positions of the layers along the *z* direction. The fits to the data with a function *C_z_*(*r*)/*C_z_*(0) = 1 − *ar*^θ^, where *a* and θ as the fitting parameters, are shown in solid, dashed, and dotted lines. The fitting parameters for the data shown are the following: for *z* = 3 μm, *a* = 0.256, θ = 0.597; for *z* = 5 μm, *a* = 0.315, θ = 0.752; for *z* = 7 μm, *a* = 0.309, θ = 0.790. (Inset) Variation of ξ*_z_* with *z* for the spatial patterns of NTHI in panel A. dpi, days postinfection. (D) Variation of *C_z_*(*r*)/*C_z_*(0) with *r* for the spatial patterns generated by the NTHI in the CLSM images in panel B. The data are fitted with the same function as in panel C. The fitting parameters are the following: for *z* = 3 μm, *a* = 0.464, θ = 0.574; for *z* = 5 μm, *a* = 0.464, θ = 0.567; for *z* = 7 μm, *a* = 0.474, θ = 0.558. (Inset) Variation of ξ*_z_* with *z* for the spatial patterns of NTHI in panel B. (E) ξ*_z_* represents a typical size of a cluster of NTHI in the CLSM images in panels A and B.

NTHI formed biofilms in the middle ears of Chinchilla lanigera. Sections of the middle ear epithelium and associated biofilms 4 and 11 days after challenge were cut, immunolabeled for NTHI and extracellular DNA (eDNA), and then imaged using confocal laser scanning microscopy (CLSM) (Fig. 1A and B). Further details regarding the experiments can be found in reference 4. The green and blue colors show the NTHI bacterial cells and the DNA strands in the images, respectively. On visual inspection, the NTHI aggregates appeared to be 1 to 3 μm in diameter. The size of the NTHI aggregates is characterized quantitatively later in this section. The spatial organization of the NTHI bacterial cells within the biofilm was characterized by analyzing the pair correlation function *C_z_*(*r*). *C_z_*(*r*) was calculated using the densities (or {ρ(*x*,*y*,*z*)}) of the NTHI bacterial cells at locations {(*x*,*y*,*z*)} within the biofilm. We assumed ρ(*x*,*y*,*z*) to be proportional to the image intensity at the location (*x*,*y*, *z*) in the CLSM image. *C_z_*(*r*) is defined as *C_z_*(*r*) = 〈1/*L*^2^ ∑*_x_*_,_*_y_* ρ(*x*,*y*, *z*)ρ(*x* + *r_x_* , *y* + *r_y_* , *z*)〉 − 〈1/*L* ∑*_x_*_,_*_y_* ρ(*x*,*y*,*z*)〉 〈1/*L* ∑*_x_*_,_*_y_* ρ(*x*,*y*,*z*)〉, where *L* is the length of the sample along the *x* or *y* direction. The above definition of *C_z_*(*r*) assumes translation symmetry and rotational invariance of the bacterial density, and 〈…〉 indicates an average over an ensemble of density configurations. The behavior of *C_z_*(*r*) at length scales larger than the scale of image resolution (*a*_0_ ≈ 0.29 μm) and smaller than an intermediate scale (*w* ∼ several microns) can indicate the presence of specific spatial patterns within the range as we briefly mention below. Further details regarding the analysis can be found in reference 7. When the length scale *r* is within the range, *a*_0_ ≤ *r* ≤ *w*, *C_z_*(*r*) can be approximated as *C_z_*(*r*)/*C_z_*(*r* = 0) ≈ 1 − *ar*^θ^ (26, 27). The less than unity values of exponent θ indicate the presence of clusters with fractal surfaces (27–29). The variation of *C_z_*(*r*)/*C_z_*(0) at distances *r* ≤ 1 μm for the NTHI biofilms at day 4 and day 11 postchallenge shows a behavior consistent with 1 − *ar*^θ^, where θ < 1 indicates the presence of fractal interfaces in the *in vivo* biofilm (Fig. 1C and D). The fit to the pair correlation data [*C_z_*(*r*)/*C_z_*(*r* = 0) versus *r*] at small values of *r* (*r* = 0 to *r* = 1.5 μm) with the function 1 − *ar*^θ^ (θ < 1) was compared with a fit with a function 1 − *ar* using corrected Akaike information criterion (AIC_c_) (see Text S1, Table S1A, and Fig. S1 in the supplemental material). In addition, the ability of an exponential decay function [exp(−*ar*)] to describe the data was compared with that of a nonexponential decay function exp(−*ar*^θ^) using AIC_c_ (Text S1, Table S1A, and Fig. S2). In both the above comparisons, the data convincingly supported the nonlinear and the nonexponential fits. A piece-wise fitting function with two different θ exponents (θ = θ_<_ for *r* < *r_c_* and θ = θ_>_ for *r* > *r_c_*) fitted the pair correlation over a longer range of *r* from *r* = 0 to *r* = 5 μm (Fig. S2). This demonstrates that the behavior of *C_z_*(*r*) indicates the presence of fractal interfaces in the biofilm. *C_z_*(*r*) also provides estimates of characteristic length scales (e.g., size of a bacterial cluster or a void region) present in the spatial organization. One such characteristic length scale ξ*_z_* (Fig. 1E) can be defined using *C_z_*(*r* = ξ*_z_*)/*C_z_*(0) = ½. The ξ*_z_* values for the images in Fig. 1A estimate the sizes of the NTHI clusters. The ξ*_z_* values for the 4-day-old and 11-day-old biofilms (Fig. 1C and D, insets) are less than 3 μm, which are substantially smaller than the large ξ*_z_* values (∼20 μm) obtained in NTHI biofilms formed *in vitro* (7).

10.1128/mSphere.00254-19.1 TEXT S1 Comparison of different fits to the pair correlation data. Download Text S1, PDF file, 0.08 MB. Copyright © 2019 Brown et al. 2019 Brown et al. This content is distributed under the terms of the Creative Commons Attribution 4.0 International license.

10.1128/mSphere.00254-19.2 FIG S1 Fits to the *C_z_*(*r*)/*C_z_*(0) versus *r* data for day 4 and day 5 biofilms using the function 1 – *ar* (A and B) or 1 – *ar*^θ^ (C and D). The data composed of 15 data points from *r* = 0 to *r* = 1.5 μm are fit with the functions. The fits and the data are displayed by solid or dashed lines and with symbols, respectively. The fits to the data are shown in the same colors as the data. Download FIG S1, PDF file, 0.2 MB. Copyright © 2019 Brown et al. 2019 Brown et al. This content is distributed under the terms of the Creative Commons Attribution 4.0 International license.

10.1128/mSphere.00254-19.3FIG S2Fits to the log[*C_z_*(*r*)/*C_z_*(0)] versus *r* data for day 4 and day 5 biofilms using the function –*ar* (A and B) or –*ar*^θ^ (C and D) or a crossover function (E). The data composed of 15 data points from *r* = 0 to *r* = 1.5 μm are fit with the functions. The fits and the data are displayed in solid or dashed lines and with symbols, respectively. The fits to the data are shown in the same colors as the data. (E) Fit to the *C_z_*(*r*)/*C_z_*(0) versus *r* data from *r* = 0 to *r* = 5 μm for the 11-day-old biofilm with a crossover function that fits the data with 1 − *ar*^θ^ with θ = θ_<_ for *r* < *r_c_* and θ = θ_>_ for *r* > *r_c_*. The fits to the data (shown with symbols of different colors) are displayed with solid lines. The fitting function was proposed in reference 7 and is given by $$f(r)=1-\left(1-\frac{r^{{}^{5}}}{r^{5}+r_{c}{}^{5}}\right)a_{<}r^{\theta_{<}}+\frac{r^{{}^{5}}}{r^{5}+r_{c}{}^{5}}a_{>}r_{\theta_{>}}$$ where $r={\left(a/a_{<}\right)}^{\theta_{<}-\theta_{>}}$. We obtained the following values for the fitting parameters: for *z* = 3 μm, *r_c_* = 1.07 μm, *a*_<_ = 0.415, θ_<_ = 0.464, *a*_>_ = 0.544, θ_>_ = 0.226, AIC_c_ = −1,096.8; for *z* = 5 μm, *r_c_* = 1.05 μm, *a*_<_ = 0.415, θ_<_ = 0.458, *a*_>_ = 0.540, θ_>_ = 0.251, AIC_c_ = −1,047.9; for *z* = 7 μm, *r_c_* = 1.05 μm, *a*_<_ = 0.427, θ_<_ = 0.458, *a*_>_ = 0.546, θ_>_ = 0.263, AIC_c_ = −1039.0. Download FIG S2, PDF file, 1.5 MB.Copyright © 2019 Brown et al.2019Brown et al.This content is distributed under the terms of the Creative Commons Attribution 4.0 International license.

10.1128/mSphere.00254-19.9 TABLE S1 (A) Comparison of fits to the *C_z_*(*r*)/*C_z_*(0) versus *r* data with fitting functions 1 – *ar* and 1 – *ar*^θ^, respectively. (B) Comparison of fits to the log(*C_z_*(*r*)/*C_z_*(0)) versus *r* data with fitting functions –*ar* and –*ar*^θ^, respectively. Download Table S1, PDF file, 0.10 MB. Copyright © 2019 Brown et al. 2019 Brown et al. This content is distributed under the terms of the Creative Commons Attribution 4.0 International license.

### Development of an agent-based model.

**FIG 2:**
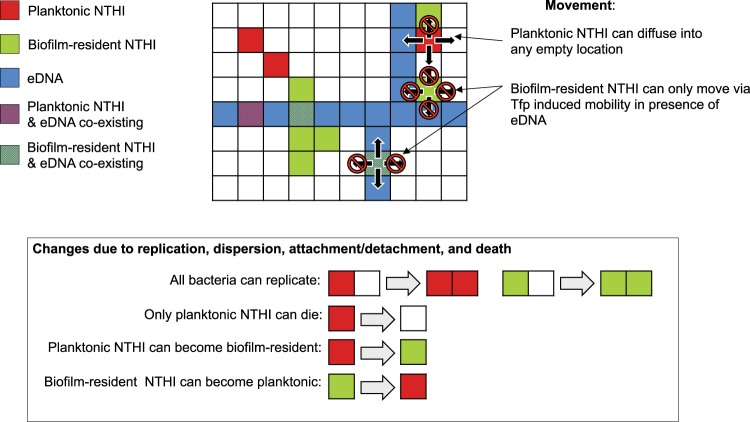
A schematic depiction of the agent-based model. The potential changes in a representative biofilm configuration due to the processes considered in the agent-based model are shown schematically. The checkered box shows a typical biofilm configuration in a *z* plane in the model. An individual voxel is either unoccupied (white) or is occupied with a planktonic (maroon) or a biofilm-resident (green) NTHI or extracellular DNA (blue). The planktonic (purple) or biofilm-resident bacteria (cyan) can coexist in a voxel with extracellular DNA. Each voxel can be occupied by at most one bacterial cell. The physical movements of the NTHI due to diffusion or Tfp-induced motility are shown with black arrows. The prohibited movements (e.g., diffusion of planktonic NTHI to a neighboring voxel occupied by another NTHI) are marked with null symbols. The processes that arise due to NTHI replication, NTHI dispersion, attachment (or detachment) of the NTHI to (or from) the ECM, and NTHI death are depicted in the rectangular box below.

We developed a spatially resolved agent-based model to describe the formation of NTHI biofilms in the chinchilla middle ear. The model was based on the agent-based model that described the formation of NTHI biofilms in two dimensions in *in vitro* culture wells (7). The key elements of the model are described below. The processes involved in the model are shown schematically in Fig. 2. The parameter values are shown in Table 1, and further details regarding the model simulations are detailed in Materials and Methods and in the supplemental material. In the model, the space is discretized into cubic “voxels” of size *l*_0_ (=0.5 μm) such that each voxel can possess at most one NTHI bacterium. The NTHI bacterial cells can be either in the biofilm-resident state or in the planktonic state. The biofilm-resident (or planktonic) bacterial cells are assumed to be attached (or unattached) to extracellular DNA and other (not explicitly modeled) molecules that are part of the extracellular matrix (ECM) that comprises the biofilm. Epithelial cells are also not explicitly included in the model.

**TABLE 1:** Parameters used in the agent-based model^*a*^

Symbol	Meaning	Unit	Value or range	Comment(s)
*N_x_*	No. of voxels in the *x* direction		100	
*N_y_*	No. of voxels in the *y* direction		100	
*N_z_*	No. of voxels in the *z* direction		100	
*l*_0_	Length of a voxel’s side	μm	0.5	The volume of each voxel is approximately that of one NTHI bacterium (7).
*k*_plank_	Rate of diffusive movements for the planktonic bacteria	1/s	2.4	Calculated using *k*_plank_ = 6*D*_plank_/*l*_0_^2^, where *D*_plank_ = 0.2 μm^2^/s and is the diffusion coefficient for the planktonic bacteria (7).
*k*_twitch_	Rate of ballistic movement of NTHI on extracellular DNA strands induced by Tfp-mediated twitching	1/s	0.12	Calculated using, *k*_twitch_ = υ_twitch_/*l*_0_, where υ_twitch_ is the linear velocity of attached bacteria moving on the eDNA network. Estimated to be similar to P. aeruginosa as studied in reference 30.
*k*_turn_	Rate of attached bacteria changing direction	1/s	0.02	Estimated to be similar to P. aeruginosa as studied in reference 30. The half-life of velocity autocorrelation is about 30 s, this gives a rate constant of ln(2)/30 ≈ 0.023 s^−1^
*k*_div_	Rate of division per nutrient density (for both attached and planktonic bacteria)	μm^3^/s	0.0003	Assuming 1 division per hour at a nutrient density of 1/μm^3^
*k*_death_*	Rate of planktonic bacterial death	1/s	10^−3^−10^−1^	Assuming faster than cell division, slower than movement
*k*_attach_*	Rate of planktonic bacteria becoming attached	1/s	10^−4^−10^−2^	Assuming faster than cell division, slower than movement
*k*_detach_*	Base rate of attached bacteria becoming planktonic	1/s	10^−5^−10^−3^	Assuming similar to *k*_div_
*k*_quorum_*	Additional rate of attached bacteria becoming planktonic, when the quorum sensing mechanism is activated	1/s	10^−3^−10^−1^	Assuming >> *k*_div_
*d*_quorum_*	(Manhattan) distance (in voxels) away to use in the local density calculation for quorum sensing	Δ*l*	1−4	Sets the size of bacterial clusters
*n*_quorum_*	Threshold to activate quorum sensing		25–75%	Total bacterial density within *d*_quorum_ of a bacterium
*k*_nutrient_*	Rate of nutrient generation	1/(μm^3^s)	10^−7^−10^−5^	Chosen to create a nutrient-starved state
*n*_max_*	Maximum nutrient density	1/μm^3^	0.05−0.25	Chosen to create a nutrient-starved state

a Parameters marked with an asterisk were optimized within the range shown. If the range is given in powers of 10, the optimization was done on a logarithmic scale.

Bacterial growth/division occurs at a rate proportional to the density of nutrient in the simulation box; each division event decreases the amount of nutrient. Nutrition is modeled at a global level where there is a source of new nutrient into the simulation box with a specified carrying capacity. In the simulation, the amount of nutrient in each voxel increases at every time step until it reaches a maximum value *n*_max_, for each bacterial division nutrient amount in each of the voxels is decreased by 1/*V*, where *V* is the volume of the simulation box. The extracellular DNA strands are assumed to generate a mesh-like structure that was fixed at the beginning of the simulation. Based on experimental images, a rough approximation for the eDNA was used: parallel DNA strands (going along the *x* direction) that are 1 μm thick and spaced by 5 μm, there are some random breaks in the strands (5% probability every 5 μm) and there are random bridges between strands in the plus or minus *y* and *z* directions (5% probability for each bridge every 5 μm). This assumption was implemented to investigate the role of the eDNA mesh structure in affecting the biofilm morphology. The assumption ignores the details regarding the formation of extracellular DNA mesh, as the biofilm forms within the host and the extracellular DNA strands are produced by NTHI. Since precise molecular details regarding the contribution of the extracellular DNA generated by the NTHI and the dead cells are not well-known, we avoided modeling these processes explicitly to protect the model from overparameterization.

We modeled the following processes in our model.

### (i) NTHI movements.

Three different processes produce movement of NTHI bacterial cells in our model. (i) When the number of NTHI in a voxel exceeds a maximum packing number (*n*_thres_ = 1 bacterial cell), the excess bacteria are moved to an adjacent compartment that can accommodate the excess bacteria without going over the threshold *n*_thres_. This rule represents passive movement of NTHI due to the mechanical forces exerted by the neighboring bacteria in a tightly packed region. (ii) In our simulation, the biofilm-resident bacteria on extracellular DNA can “twitch” to move along the extracellular DNA network. This rule was constructed motivated by observation of twitching motility of P. aeruginosa on extracellular DNA tracks in phase-contrast imaging experiments (30). However, such movements have not yet been directly observed for NTHI. Previous experiments showed that NTHI exhibits twitching motility on solid or biotic surfaces which is not included in the current model. Therefore, the movement of NTHI modeled here (twitching along eDNA strands) is a hypothesis used in the *in silico* model. This movement is velocity driven and nondiffusive (ballistic) on short time scales and always moves in a direction with extracellular DNA strands (30). (iii) Planktonic NTHI bacterial cells can move diffusively between the voxels.

### (ii) NTHI dispersion.

Quorum signaling brings the bacteria together and induces dispersion of the bacterial cells. NTHI dispersion changes biofilm-resident NTHI bacterial cells to planktonic bacterial cells. The dispersion is activated in a voxel when the number of NTHI bacterial cells in neighboring voxels (at a range of *d*_quorum_) reaches a threshold (*n*_quorum_). This step approximates and effectively models dispersion (at a rate *k*_quorum_) arising due to attaining a threshold number of quorum-sensing-mediating molecules (modeled implicitly) that are secreted by NTHI in the local neighborhood.

### (iii) Attachment/detachment of NTHI bacterial cells.

Biofilm-resident (or planktonic) NTHI bacterial cells can detach (or attach) from (or to) the ECM to become planktonic (or biofilm-resident) bacterial cells.

### (iv) Elimination of NTHI cells by the host response.

The multifactored host response, including mucociliary clearance, antimicrobial proteins, neutrophil extracellular trap (NET) formation, nutritional immunity, and the adaptive immunity that eliminates the NTHI is not explicitly modeled. We represented the effect of the host response by removal of the planktonic NTHI bacterial cells at a higher rate compared to their replication rate. The elimination of the biofilm-resident NTHI cells by the host response was assumed to occur at a much lower rate and was ignored in the model.

### (v) Simulation of the model with time.

The model is progressed in time by executing the above processes with specific probabilities following a standard rejection kinetic Monte Carlo (KMC) algorithm (7). The parameter values used in the model are shown in Table 1. Further details are provided in Materials and Methods.

### (vi) Estimation of model parameters.

Parameters marked with asterisks in Table 1 are estimated using the *C_z_*(*r*) calculated from the CLSM images. We use a particle swarm optimization to find the best-fit parameter values. Details are provided in Materials and Methods.

### Agent-based model describes the organization of NTHI bacterial cells *in vivo* statistically.

**FIG 3:**
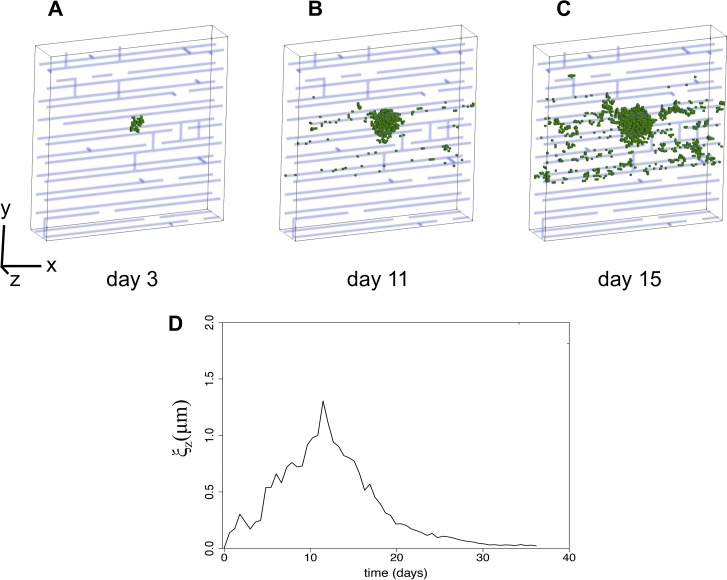
Kinetics of spatial pattern of biofilm-associated NTHI in the agent-based model shows growth of bacterial clusters followed by bacterial dispersal. (A to C) Typical configuration of biofilm-resident NTHI bacterial cells (green voxels) in the agent-based model at day 3 (A), day 11 (B), and day 15 (C). The mesh formed by the extracellular DNA is shown in blue. The rectangular box displays a region of dimensions 50 μm (*x* direction) by 50 μm (*y* direction) by 10 μm (*z* direction). The parameter values for the simulations are shown in Table 1, and Table 2 shows the best-fit parameters to the CLSM image obtained at 11 days postchallenge. (D) Change in the correlation length ξ_z_ calculated from a slice though the center of the box ±1 μm (where the bacteria were initialized), with time for the model parameter values associated with the configurations in panels A to C.

The agent-based model was used to generate spatial organization of NTHI bacteria in three dimensions at different days postchallenge. The simulation was initiated by the introduction of a few (∼2) NTHI in two voxels within the simulation box of size 50 × 50 × 50 μm^3^. These bacteria replicate and migrate following the processes described above and generate clusters of biofilm-resident NTHI bacterial cells. The size of the cluster increases initially. However, as the size of the cluster increases, the effect of the quorum-sensing mechanism is activated in the model, increasing the dispersion of biofilm-associated bacteria to planktonic bacteria. Most of the planktonic bacteria are removed by the simulated effective host response. However, a small fraction of these bacteria become reattached to the ECM and generate new clusters of biofilm-resident NTHI. The hypothesized twitching movements of the NTHI on the strands of extracellular DNA mesh also spread out the biofilm-resident bacterial cells. Therefore, the size of the NTHI clusters start decreasing after initial growth, as many smaller clusters form and the system is no longer dominated by a single large cluster (Fig. 3A to C). The biofilm-resident NTHI bacterial cells settle into such configurations for a very long time, and the system appears to have reached a more spatially inhomogeneous steady state (Fig. S4 and Movie S1). The above kinetics can be characterized by the initial increase followed by a decrease and then saturation to a fixed value in the correlation length, ξ*_z_*(*t*) (Fig. 3D). The values of the cluster sizes [or ξ*_z_*(*t*)], the timing of its peak value, and the cluster size at long times depend on the values of the parameters in Table 1, though the qualitative nature of above-described changes remain the same for wide ranges of these parameter values.

10.1128/mSphere.00254-19.10 MOVIE S1 Time course of the spatial organization of the NTHI (green voxels) in the biofilm in the agent-based model. The size of the displayed simulation box is the same as in Fig. S1, and the extracellular DNA strands are shown in blue. Each frame is increased by 3 days, and the movie shows the time course from day 1 to day 36. The parameter values for the simulation are the same as in Fig. 3 in the main text. Download Movie S1, AVI file, 5.9 MB. Copyright © 2019 Brown et al. 2019 Brown et al. This content is distributed under the terms of the Creative Commons Attribution 4.0 International license.

We conducted a simulation of the model without any DNA strands. Since in the model we stipulated that NTHI twitch only along the DNA strands, this twitching motility will be absent in the model when DNA strands are not present. In the simulation that did not contain any extracellular DNA, the bacterial cells were less spread out compared to the simulation that contained extracellular DNA strands (Fig. S3). There are two important points to note here. (i) We reiterate that previous work has shown that NTHI exhibits twitching motility on both biotic and abiotic surfaces, and there is no biological evidence that NTHI twitching motility requires the presence of extracellular DNA. The twitching motility along the extracellular DNA in the model is based on data from studies with P. aeruginosa, but such movements have not yet been directly observed for NTHI. (ii) In the simulations without any DNA strands, we found that the bacterial cells did not spread out and form smaller clusters in large numbers as can be seen in the simulation with DNA strands. Note that both the DNA strands are exported and that the Tfp are expressed through the ComE-comprised pore in the outer membrane of NTHI, and thereby, a *comE* mutant of NTHI will lack both the Tfp-induced twitching activity and the ability to export DNA strands (8). Thus, the modification of the model where DNA is absent from the biofilm and the bacteria are unable to execute any twitching movements (which occurs as a consequence of the model assumption stated above) might appear to describe biofilm formation by the *comE* mutant as a consequence of these linked biological activities. However, we have not yet tested the model predictions against any *in vivo* experiments.

10.1128/mSphere.00254-19.4 FIG S3 Organization of bacterial cells (green voxels) at day 4 (A) and day 11 (B) for a simulation of the model carried out without any extracellular DNA strands. The rest of the parameters are the same as in Table 2. (C) Snapshot of bacterial cells at day 4 when the simulation was carried out in the presence of extracellular DNA strands (blue). The parameters for the simulation are given in Table 2 (day 4 best fit). The above snapshots show lower spreading of bacterial cells outside the central cluster compared to the case with extracellular DNA as can be seen in the snapshot of bacterial cells in panel C and in the 11 day snapshot in Fig. 3B. Download FIG S3, PDF file, 0.2 MB. Copyright © 2019 Brown et al. 2019 Brown et al. This content is distributed under the terms of the Creative Commons Attribution 4.0 International license.

10.1128/mSphere.00254-19.5 FIG S4 The NTHI in the model show spatially inhomogeneous organization at late times. A typical configuration of simulated biofilm-resident NTHI (green voxels) in the agent-based model at day 36 is shown. The extracellular DNA strands are shown in blue. The size of the volume shown is the same as in Fig. 3A. The parameters used for the simulation are the same as in Fig. 3A to C in the main text. The simulations generated by the agent-based model appear to have reached a steady state by day 36 for the above parameter values. This shows that bacteria can reside within the host environment long-term by organizing in spatial patterns that balance opposing effects (e.g., replication, dispersion versus elimination of planktonic bacterial cells) toward the survival of bacteria. Download FIG S4, PDF file, 0.4 MB. Copyright © 2019 Brown et al. 2019 Brown et al. This content is distributed under the terms of the Creative Commons Attribution 4.0 International license.

### Estimation of parameters not available from experiment.

**FIG 4:**
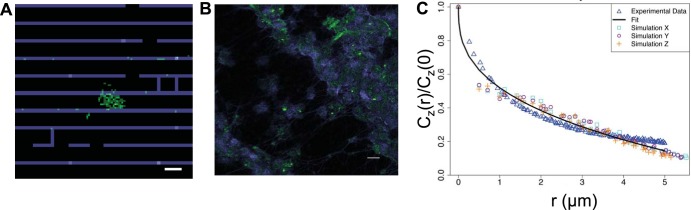
Spatial pattern of NTHI in the CLSM image of the biofilm formed by NTHI in the chinchilla middle ear and the agent-based model show similar fractal structure. (A) A representative configuration in the agent-based model for a section through the middle of the simulation box (±1 μm) showing NTHI (green) and the mesh formed by extracellular DNA strands (blue). The configuration is produced by simulation of the model for the parameter values that generate the best fit between the model and the data obtained from the CLSM images at day 11 postchallenge. Bar = 5 μm. (B) NTHI (green) and the extracellular DNA strands (blue) in the CLSM image for a single z-stack for the biofilm obtained from the chinchilla middle ear at day 11 postchallenge. Bar = 10 μm. (C) Comparison between the pair correlation function [*C_z_*(*r*)/*C_z_*(0) versus *r*] calculated using the CLSM image data (*z* = 4 μm) shown in panel B (blue triangles) for the biofilm formed in the chinchilla middle ear at day 11 postchallenge and the configuration generated by agent- based model (box, circle, and plus signs) at the best-fit parameter values (Table 2). The solid black line shows the best fit to the *C_z_*(*r*)/*C_z_*(0) versus *r* data calculated from the CLSM image. The pair correlation for the agent-based model was fitted at the midpoint along the *x* direction (box), *y* direction (circle), or the *z* direction (+) to account for the uncertainty in matching the orientations of the biofilms formed *in vivo* with that generated by the agent-based model.

We estimated parameter values for eight different parameters (indicated with an asterisk in Table 1) whose values are not known from laboratory measurements by fitting the pair correlation function *C_z_*(*r*) for a specific *z* value at a particular day postchallenge (e.g., 11 days) that is calculated from the NTHI bacterial cell configurations in the agent-based model with the *C_z_*(*r*) calculated from the corresponding CLSM image. Since there was no reference to match the *z* value between the *in silico* and *in vivo* configurations, we arbitrarily chose to fit the NTHI bacterial cell configurations in the center z-stack (e.g., *z* = *L*/2) of the CLSM image. The arbitrariness in choosing the *z* value for comparison does not affect the general conclusions drawn from the parameter estimations, as ξ*_z_*(*t*) values were quite similar between different z-stacks in the *C_z_*(*r*) calculated from the CLSM images (Fig. 1C, inset). The particle swarm optimization method obtained a low value for the cost function (*E*) in equation 2, indicating an excellent fit for the NTHI bacterial cell organization at 11 days postchallenge. Since we fit the pair correlation function with the *in vivo* images, the agreement refers to statistically similar NTHI bacterial cell configurations between the model and the NTHI bacterial cell configuration in the chinchilla middle ears. The representative *in silico* and *in vivo* configurations are shown in Fig. 4A and B. The fits to the *C_z_*(*r*) are shown in Fig. 4C. The estimated parameter values and the standard deviations of those values are shown in Table 2.

**TABLE 2:** Best-fit model parameters to experimental images resulting from particle swarm optimization

Symbol	4-day optimal value	11-day optimal value
log_10_*k*_death_	−1.38 ± 0.20	−1.53 ± 0.27
log_10_*k*_death_	−2.82 ± 0.61	−4 ± 0.07
log_10_*k*_death_	−5.78 ± 0.18	−5.80 ± 0.08
log_10_*k*_quorum_	−4 ± 0.11	−2.55 ± 0.40
*d*_quorum_	4 ± 0.41	2 ± 0.32
*n*_quorum_	51.9% ± 2.9%	28.0% ± 1.0%
log_10_*k*_nutrient_	−5.33 ± 0.55	−7 ± 0.04
*n*_max_	0.104 ± 0.013	0.05 ± 0.0003

The rates in Table 2 show changes in the simulation parameters as the infection progresses from day 4 to day 11. The rate of elimination of the planktonic NTHI (or *k*_death_) by the host response as well as the rate of dispersion (or *k*_quorum_) induced by quorum sensing increase from day 4 to day 11, increasing the resistance of the host response on NTHI growth on the later days. This mechanism is consistent with the slight decrease (from ∼1 μm to ∼0.5 μm) of the NTHI spatial cluster sizes as the NTHI biofilm age increases from day 4 to day 11 (Fig. 1). We note the following caveat in the above parameter estimation. The precise values of the parameters appear to depend on the choice of the initial bacterial organization. For example, when a biofilm configuration obtained at day 4 for a model that fit the 4-day-old biofilm data was further simulated using the parameters obtained for the fit to the 11-day-old biofilm (Table 2), the biofilm configurations in the simulation at day 11 (Fig. 3B) agreed qualitatively with the data at day 11 (Fig. S5) in that the clusters of bacterial clusters become more diffuse, and the difference in the amount of bacteria between Fig. 3B and Fig. S5 is due to the different initial conditions. However, the behavior of the pair correlation function did not match with that obtained from the data well (Fig. S6A). The calculation of two-time autocorrelation function (31), *A*(*t*_1_,*t*_2_) = 〈ρ(*r*,*t*_1_)ρ(*r*,*t*_2_)〉, where *t*_2_ > *t*_1_, for these simulations seemed to indicate a power law decay (*A*(*t*_1_,*t*_2_)∝ 1/(*t*_2_)^λ^ f(*t*_1_,*t*_2_)) with time (Fig. S6B). The power law decay of the autocorrelation function points to a strong dependence of the system kinetics on the initial condition which has been observed in many models of ordering kinetics in statistical mechanics (31). However, the mechanisms that underlie the initial condition dependence of the spatial kinetics in our model are currently unclear and need further investigation.

10.1128/mSphere.00254-19.6 FIG S5 Organization of bacterial cells (green voxels) at day 11 when the stimulations started at day 4 with a bacterial configuration obtained from the model that was fit to the 4 day biofilm data. The simulation was carried out using the parameters (Table 2) that generated the best fit to the 11 day biofilm data as described in the main text. The extracellular DNA mesh is shown in blue. Download FIG S5, PDF file, 0.1 MB. Copyright © 2019 Brown et al. 2019 Brown et al. This content is distributed under the terms of the Creative Commons Attribution 4.0 International license.

10.1128/mSphere.00254-19.7 FIG S6 (A) *C_z_*(*r*)/*C_z_*(0) versus *r* calculated for configurations of bacterial cells at day 11 when the simulation was started at day 4 as described in the main text. The pair correlation does not agree well with that calculated from the CLSM images at day 11 (blue triangles). (B) Decay of the autocorrelation function *A*(*t*_1_,*t*_2_) with *t*_2_, where *t*_1_ = 4 days and *t*_2_ varies from day 4 to day 11. The autocorrelation function is defined in the main text and is calculated for the simulations described in panel A. The log(*A*(*t*_1_,*t*_2_)) versus log(*t*_2_) data (blue triangles) can be fit (solid blue) with a linear function of log(*t*_2_) (−0.774log(*t*_2_) + 0.462), which hints to a power law decay of the autocorrelation function. Download FIG S6, PDF file, 0.3 MB. Copyright © 2019 Brown et al. 2019 Brown et al. This content is distributed under the terms of the Creative Commons Attribution 4.0 International license.

## DISCUSSION

The analysis of spatial organization of NTHI in biofilms formed in chinchilla middle ears using pair correlation functions revealed the presence of surface fractal patterns at short length scales (∼1 μm). Similar fractal patterns were also found in the biofilms formed by NTHI in *in vitro* cultures (7). The presence of such surface fractal patterns indicates scale invariant organization of NTHI within the biofilm, which can potentially facilitate absorption of nutrients by increasing the surface area of the interfaces between the bacterial cells and the local environment in the nutrient-restricted middle ear. The increased surface area could also further expose the bacterial cells to antimicrobial proteins or other immune effectors. However, the ECM in the biofilm has been found to provide protection to biofilm-resident NTHI against the host immune response (9), and therefore, the survival of the NTHI *in vivo* could depend on finding an optimal pattern of spatial organization of NTHI within the biofilm that balances the above trade-offs. Our analysis also showed that the sizes of the NTHI spatial clusters are much smaller (∼1 μm) compared to their *in vitro* counterparts which can be more than 20 μm in diameter (7). As we discuss below, our modeling showed that these smaller size clusters could arise due to the elimination of planktonic NTHI bacterial cells due to the host immune response (or other means that eliminate planktonic bacteria). A similar decrease in the size of aggregates of bacterial cells in biofilm patches formed within the host by bacterial pathogens in chronic infections ranging from cystic fibrosis to rhinosinusitis compared to biofilms formed *in vitro* has also been observed (14).

We developed an agent-based model including basic processes such as NTHI replication, quorum sensing, dispersion of biofilm-resident NTHI to planktonic NTHI, and elimination of planktonic NTHI by the host immune response to describe formation of NTHI biofilms in three dimensions in the chinchilla middle ears. The model was a modification of our previously developed model in two dimensions (7) that described formation of NTHI in culture. The agent-based model incorporated most of the processes that were present in our previous agent-based model except the ones that were associated with the feeding protocol. The key modifications made in the agent-based model to describe biofilm formation *in vivo* were inclusion of the elimination of planktonic NTHI by the host immune response, no dependence of the nutrient production on physical orientations of biofilm, and creation of a nutrient-restricted environment. The model was able to reproduce the surface fractal patterns and the small sizes of NTHI clusters in the biofilms formed *in vivo* (Fig. 4). The perturbations of the model parameters showed that in the absence of the elimination of the planktonic NTHI, the NTHI bacterial cells formed large clusters and filled up the simulation box (see Fig. S7 in the supplemental material), demonstrating the key role elimination of planktonic cells (such as by the host response) plays in limiting the NTHI cluster sizes in the model. This behavior suggests the presence of the host response, which can be comprised of mucociliary clearance, antimicrobial proteins, neutrophil extracellular traps (NETs), and nutritional immunity, among others, each of which is likely important in limiting the size of the bacterial clusters in biofilms formed within the host. The agent-based model does not explicitly include the layer of epithelial cells in the middle ear, which produce antimicrobial proteins or induce additional protective host responses (e.g., mucociliary movements) that impact relative survivability of NTHI. Analyzing the roles of these processes in shaping the organization of NTHI within the biofilm can provide key mechanistic insights regarding the transport of antimicrobial proteins and antibody molecules induced by vaccine candidates. These processes will be included in future agent-based models.

10.1128/mSphere.00254-19.8 FIG S7 Spatial distribution of the NTHI when the rate of elimination of the planktonic NTHI by the host immune response was set to zero in the model. The figure shows a typical configuration of biofilm-resident NTHI (green voxels) in the agent-based model at day 11 when the rate of elimination of the planktonic NTHI by the host immune response was set to zero. The extracellular DNA strands are shown in blue. The size of the shown volume is the same as in Fig. 3A. The parameters used for the simulation is the same as in Fig. 3A to C in the main text. As shown in Fig. 3, since most planktonic bacteria are removed before they are able to nucleate new bacterial clusters, growth of new bacteria is located primarily at a central cluster for the first ∼11 days. However, in this case, all planktonic bacteria survive to nucleate new clusters, and the system quickly homogenizes to a spatially random organization of bacterial cells. Experimental images (see Fig. 1 and 4) show a system of dense clusters of bacteria, similar to what is seen in Fig. 3 and distinct with what is seen here. Download FIG S7, PDF file, 0.3 MB. Copyright © 2019 Brown et al. 2019 Brown et al. This content is distributed under the terms of the Creative Commons Attribution 4.0 International license.

We estimated several parameters in the agent-based model using the pair correlation functions calculated from the CLSM images of the NTHI biofilms formed in the chinchilla middle ears. The experimental measurements for some of these parameters were unavailable, and the rest of these parameters were associated with processes that approximated detailed molecular interactions and thus are inaccessible to direct measurements. Similar parameter estimations are common in well-mixed or spatially homogeneous models of biological processes (32, 33); however, such estimations are rare for spatial models of biofilm formation because these calculations can be computationally intensive. A recent work estimated parameters describing pairwise interactions between bacterial cells residing in biofilms formed by V. cholerae *in vitro* (25). We took advantage of parallel computation and used an optimization method (particle swarm) that can be easily parallelized to deal with this problem. Our estimations showed most of these parameters can be well estimated from the spatial information contained in *C*(*r*). The comparison of the parameters for the day 4 and day 11 CLSM images for the NTHI biofilms showed that the rate of elimination increases from day 4 to day 11; thus, the smaller size NTHI clusters at day 11 compared to those at day 4 could arise due to this effect.

In conclusion, we developed an *in silico* model for describing formation of NTHI biofilms in the chinchilla middle ears. The *in silico* model can be used to probe changes in the biofilm morphologies formed by NTHI over time *in vivo* and relate those changes to biologically relevant parameters (e.g., immune response, quorum sensing). In addition, the model can be used to test therapeutic strategies for disrupting biofilms and/or preventing their formation, e.g., increase the rate of NTHI elimination by increasing the NTHI dispersion rate from the biofilm or preventing them from entering into biofilm residence.

## MATERIALS AND METHODS

### Simulation of the agent-based model.

The model was progressed in time using a three-dimensional generalization of the standard rejection kinetic Monte Carlo (KMC) scheme described in reference 7. The time constant for the rejection KMC algorithm is based on the maximum rate in the system, *k*_max_, which in this case is *k*_plank_. Each KMC trial time is advanced by Δ*t* = −log(*u*)/4*N*_bact_*k*_max_ where *u* is a uniform random number in the interval (0,1] that is generated each MC trial. The factor 4 in the denominator of the above expression results from the number of possible processes (movement, growth, death/turning, attachment). For the chosen parameters above, on average, 0.0521 s passes every time all bacteria in the system attempt one (randomly chosen) process one time. At each KMC trial, the nutrient density in a voxel goes up by Δ*n*, given by

$$\text{Δ}n=k_{\text{nutrient}}\left(1-\frac{n}{n_{\text{max}}}\right)\text{Δ}t$$

and each time a bacterium divides (at a rate of *nk*_div_), the nutrient in all the voxels decrease by 1/*V*, where *V* is the volume of the box.

### (i) Initial condition.

All the simulations are started by having two biofilm-attached NTHI bacterial cells in the center of the domain. The extracellular DNA network is created as described above and is held fixed throughout the simulation.

### (ii) Boundary conditions.

Periodic boundary conditions along all the three spatial directions are used.

### (iii) Particle swarm optimization.

Parameters listed in Table 1 that are marked with an asterisk were optimized (on a logarithmic scale when range given as powers of 10) using that standard particle swarm optimization algorithm (34, 35) with constrains as implemented in *pyswarm* which is available at the link https://github.com/tisimst/pyswarm. The parameters used were: particle inertia coefficient of ω = 0.8, coefficient weighting a particle’s best-known position of φ*_p_* = 2.5, and coefficient weighting the swarm’s best-known position of φ*_p_* = 1.5. The swarm size was 200 particles for the 11-day fit and 400 particles for the 4-day fit (due to the shorter simulation time), and the algorithm was run for 40 iterations.

The optimization was done to match the parameters of a fit of the pair correlation function to the following equation:

(1) $$C_{z}\left(r\right)/C_{z}\left(r=0\right)=\text{1}-ar^{\text{θ}}$$

and ξ_z_ is the correlation length defined as *C_z_*(*r* = ξ_*z*_)=1/2.

The cost function used was

(2) $$E=\frac{1}{2}\underset{i=x,y,z}{\sum }\left[{\left(\frac{{\text{θ}}_{i}-{\text{θ}}_{\text{exp}}}{{\text{θ}}_{\text{exp}}}\right)}^{2}+{\left(\frac{{\text{ξ}}_{i}-{\text{ξ}}_{\text{exp}}}{{\text{ξ}}_{\text{exp}}}\right)}^{2}\right]$$

where the “exp” subscript indicates an experimental value fit from a single z-stack of an 11-day-old biofilm, and the *in silico* fits were from a 5-voxel-wide slice down the middle of the simulation box in the *x*, *y*, or *z* direction, as indicated. All three directions in the model were used for the comparison, as the orientation of the CLSM image was unknown. The sizes of the bacterial clusters in our simulations are smaller or of the same order of the smallest spacing in the eDNA mesh. The pair correlation function decays substantially across that length scale and thus does not depend on the direction. The fit parameters are shown in Table 2. The errors in the parameter values were calculated using standard deviations of points in solution space sampled with *E* < 0.01. Using the clustering algorithm in reference 36 (normalizing all parameters to a unit hypercube, and using a cutoff of 0.4), only a single cluster was found, therefore, we concluded that all points in that cluster were in the same basin. The ranges in Table 2 are the standard deviations of the locations of these points in solution space since these points give a rough approximation of the dimensions of this basin. This allowed us to distinguish between parameters that are similar and dissimilar.

### Experiments.

Chinchilla lanigera were challenged transbullarly with 2,500 CFU NTHI strain 86-028NP as described previously (4). Four or 11 days later, the bullae, which contained biofilms formed by NTHI, were removed, embedded in OCT, and snap-frozen over liquid nitrogen. After removal of the exterior bone, we cut serial 10-μm sections of the middle ear epithelium and associated biofilms, immunolabeled them for NTHI with anti-NTHI outer membrane protein (OMP), and counterstained with 4′,6′-diamidino-2-phenylindole (DAPI) to label eDNA. Samples were imaged by CLSM. Further details are provided in reference 4.
