# Supplementary material for: In Silico Modeling of Biofilm Formation by Nontypeable Haemophilus influenzae In Vivo
Source: mSphere. 2019 Jul 31;4(4):e00254-19. doi: 10.1128/mSphere.00254-19 (PMC6669334; doi:10.1128/mSphere.00254-19)
Supplement: TEXT S1 [file mSphere.00254-19-s0001.pdf]

Supplementary Text : Comparison of different fits to the pair-correlation data

We fitted the data for $C_z(r)/C_z(0)$ vs r with the following functions: i)1-*ar* (*a* is the fitting parameter), ii) $1\text{-}ar^{\theta}$ (*a* and θ are the fitting parameters, iiii) exp(-*ar*) (*a* is the fitting parameter), iv) $\exp(-ar^{\theta})$ (*a* and θ are the fitting parameters). For the functions in (iii) and (iv) the fitting was carried out in the semi-log space ($\log(C_z(r)/C_z(0))$ vs r). The first 15 data points (from r=0 to r=1.5μm) in $C_z(r)$ were used in estimating the fitting parameters. The fitting was carried out using the nonlinear least squares (nls()) function in the R statistical package. We computed the sum of the square residuals (*SSR*) and evaluated the corrected Akaike Information Criterion (AIC_c) defined as, $AIC_c = 2m + n \ln(SSR/n) + 2m(m+1)/(n-m-1)$[1], where *m* is the number of fitting parameters and *n* (=15) is the number of data points used in the fits. The difference of the AIC_c ($(\Delta AIC_c)_i = (AIC_c)_i - (AIC_c)_{min}$) between a pair of fitting functions (denoted as *i* and *j*, where function or model *j* possesses the minimum AIC_c value $(AIC_c)_{min}$ in the pair *i,j*) provides an information theoretic way to discriminate between the ability of these functions to describe the data. The AIC_c values can be used to rank the models in their ability to describe the data[1], e.g., the model corresponding to the lowest AIC_c is best model compared to the other models. If the competing model has an AIC_c value higher than <2 points compared to the best model, the competing model cannot be discriminated well from the best model; if the competing model has an AIC_c value above 9-11 or above 20, the model has little support or has no support. The following tables (Table S1) show the fitting functions and the corresponding AIC_c values. The models with the lowest AIC_c values are marked with green colored rows. The ΔAIC_c values indicate that the data convincingly support the a non-linear decay ($1\text{-}ar^{\theta}$) (Table S1A and Figure S1) or a non-exponential decay ($\exp(-ar^{\theta})$) (Table S1B and Figure S2) of the correlation function $C_z(r)/C_z(0)$. The fits to the data are shown in Figures S1 and S2.

References

1. Burnham KP, Anderson DR, Huyvaert KP. AIC model selection and multimodel inference in behavioral ecology: some background, observations, and comparisons. Behavioral ecology and sociobiology 2011;65:23-35.
