# Supplementary figures and images for: In Silico Modeling of Biofilm Formation by Nontypeable Haemophilus influenzae In Vivo
Source: mSphere. 2019 Jul 31;4(4):e00254-19. doi: 10.1128/mSphere.00254-19 (PMC6669334; doi:10.1128/mSphere.00254-19)

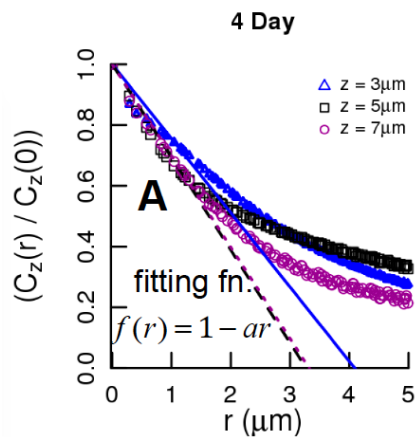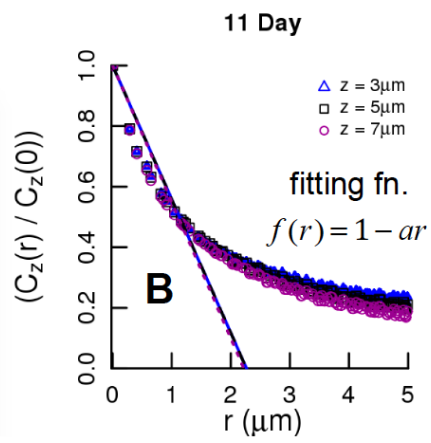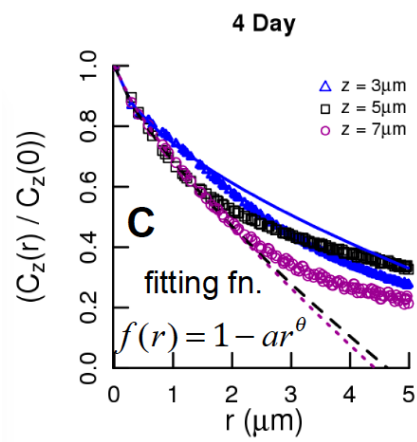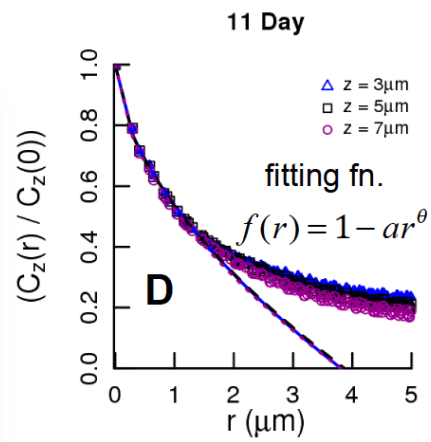

Supplement: FIG S1 [file mSphere.00254-19-sf001.pdf]

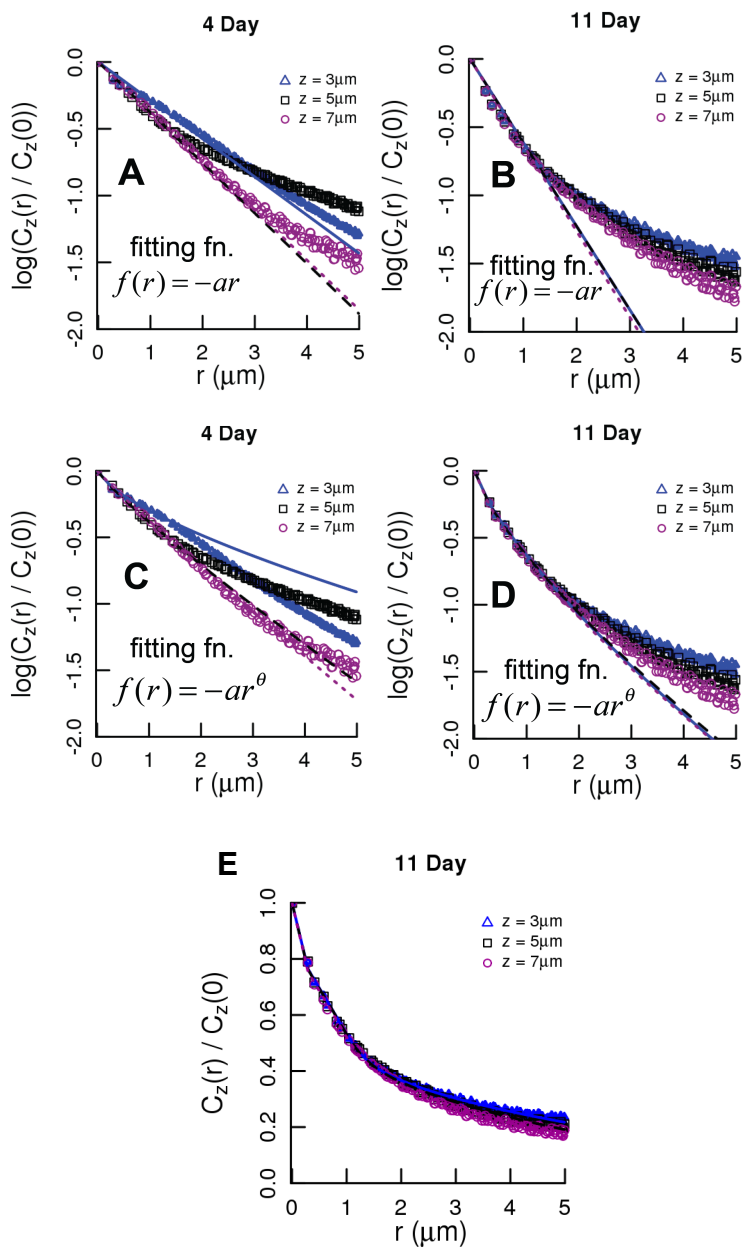

Supplement: FIG S2 [file mSphere.00254-19-sf002.pdf]

**A** 4 day

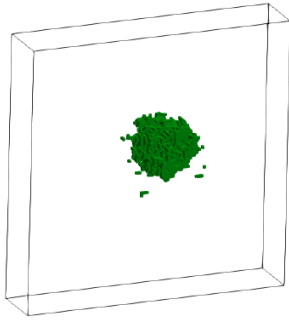

**B** 11 day

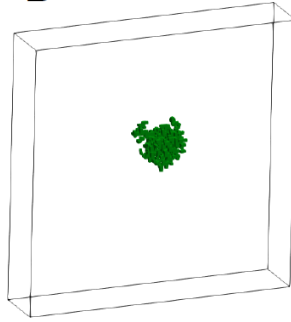

**C**

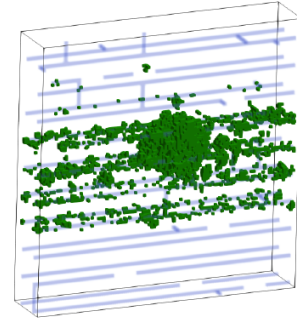

Supplement: FIG S3 [file mSphere.00254-19-sf003.pdf]

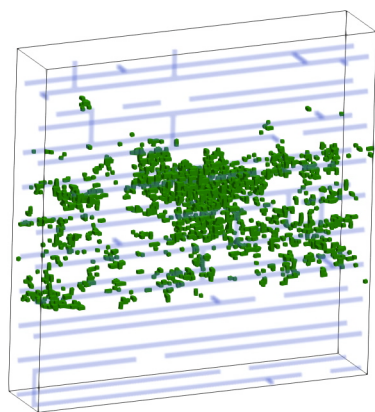

Supplement: FIG S4 [file mSphere.00254-19-sf004.pdf]

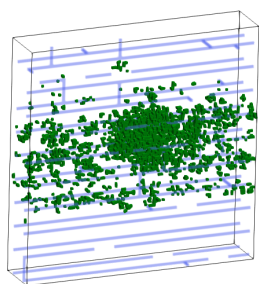

Supplement: FIG S5 [file mSphere.00254-19-sf005.pdf]

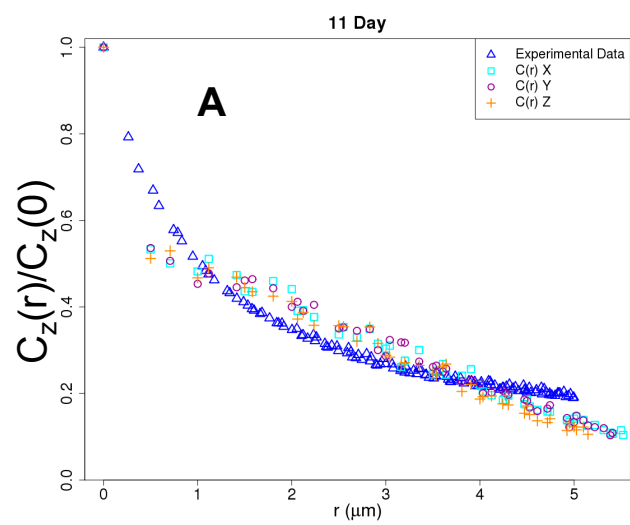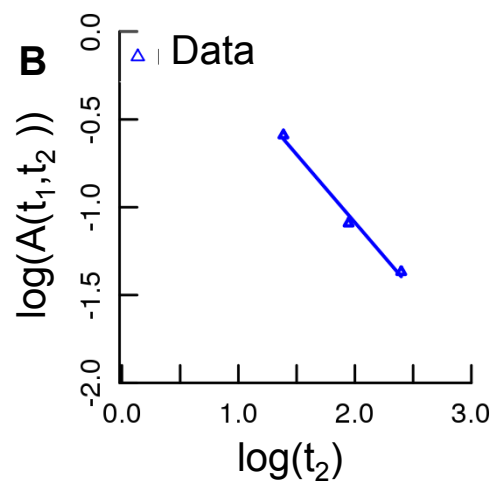

Supplement: FIG S6 [file mSphere.00254-19-sf006.pdf]

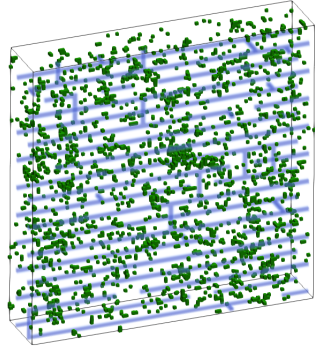

Supplement: FIG S7 [file mSphere.00254-19-sf007.pdf]
