# Supplementary material for: In Silico Modeling of Biofilm Formation by Nontypeable Haemophilus influenzae In Vivo
Source: mSphere. 2019 Jul 31;4(4):e00254-19. doi: 10.1128/mSphere.00254-19 (PMC6669334; doi:10.1128/mSphere.00254-19)
Supplement: TABLE S1 [file mSphere.00254-19-st001.pdf]

Table S1: A. Comparison of fits to the C(r)/C(0) vs r data with fitting functions 1–ar and 1–ar^{θ} , respectively.

**Data**	**Model**	**Estimated parameters**	**AIC$_c$**	**ΔAIC**
4 day old biofilm				
z=3μm	1-ar	a=0.244	-102.87519	43.01
	1-ar^{θ}	a=0.256,θ=0.597	-145.88593	
z=5μm	1-ar	a=0.306	-109.68164	29.3
	1-ar^{θ}	a=0.315,θ=0.752	-138.95889	
z=7μm	1-ar	a=0.301	-117.66818	48.5
	1-ar^{θ}	a=0.309,θ=0.790	-166.21484	
11 day old biofilm				
z=3μm	1-ar	a=0.441	-82.77731	59.1
	1-ar^{θ}	a=0.464,θ=0.574	-141.86803	
z=5μm	1-ar	a=0.441	-82.15299	57.3
	1-ar^{θ}	a=0.464,θ=0.567	-139.41435	
z=7μm	1-ar	a=0.451	-80.80226	56.1
	1-ar^{θ}	a=0.474,θ=0.558	-136.94308	

B: Comparison of fits to the log(C(r)/C(0)) vs r data with fitting functions –ar and –ar$^{\theta}$, respectively.

**Data**	**Model**	**Estimated parameters**	**AIC$_c$**	**ΔAIC$_c$**
4 day old biofilm				
z=3μm	exp(-ar)	a=0.287	-106.22926	27.7
	exp(-ar^{θ})	a=0.297,θ=0.696	-133.95785	
z=5μm	exp(-ar)	a=0.377	-124.39119	16.7
	exp(-ar^{θ})	a=0.383,θ=0.884	-141.09492	
z=7μm	exp(-ar)	a=0.371	-140.66845	3.5
	exp(-ar^{θ})	a=0.373,θ=0.951	-144.19069	
11 day old biofilm				
z=3μm	exp(-ar)	a=0.614	-91.97287	51.5
	exp(-ar^{θ})	a=0.632,θ=0.761	-143.47576	
z=5μm	exp(-ar)	a=0.613	-90.53206	52.9
	exp(-ar^{θ})	a=0.633,θ=0.750	-143.45362	
z=7μm	exp(-ar)	a=0.632	-88.76284	52.7
	exp(-ar^{θ})	a=0.652,θ=0.744	-141.48699	
